# PPARgamma in Metabolism, Immunity, and Cancer: Unified and Diverse Mechanisms of Action

**DOI:** 10.3389/fendo.2021.624112

**Published:** 2021-02-26

**Authors:** Miguel Hernandez-Quiles, Marjoleine F. Broekema, Eric Kalkhoven

**Affiliations:** ^1^ Center for Molecular Medicine, University Medical Center Utrecht, Utrecht University, Utrecht, Netherlands; ^2^ Department of Clinical Genetics, Amsterdam UMC, Vrije Universiteit Amsterdam, Amsterdam, Netherlands

**Keywords:** PPARy, adipocyte, immune cell, cancer cell, mechanism

## Abstract

The proliferator-activated receptor γ (PPARγ), a member of the nuclear receptor superfamily, is one of the most extensively studied ligand-inducible transcription factors. Since its identification in the early 1990s, PPARγ is best known for its critical role in adipocyte differentiation, maintenance, and function. Emerging evidence indicates that PPARγ is also important for the maturation and function of various immune system-related cell types, such as monocytes/macrophages, dendritic cells, and lymphocytes. Furthermore, PPARγ controls cell proliferation in various other tissues and organs, including colon, breast, prostate, and bladder, and dysregulation of PPARγ signaling is linked to tumor development in these organs. Recent studies have shed new light on PPARγ (dys)function in these three biological settings, showing unified and diverse mechanisms of action. Classical transactivation—where PPARγ activates genes upon binding to PPAR response elements as a heterodimer with RXRα—is important in all three settings, as underscored by natural loss-of-function mutations in FPLD3 and loss- and gain-of-function mutations in tumors. Transrepression—where PPARγ alters gene expression independent of DNA binding—is particularly relevant in immune cells. Interestingly, gene translocations resulting in fusion of PPARγ with other gene products, which are unique to specific carcinomas, present a third mode of action, as they potentially alter PPARγ’s target gene profile. Improved understanding of the molecular mechanism underlying PPARγ activity in the complex regulatory networks in metabolism, cancer, and inflammation may help to define novel potential therapeutic strategies for prevention and treatment of obesity, diabetes, or cancer.

## Introduction: PPARG

### General Modes of Action

**Figure 1:**
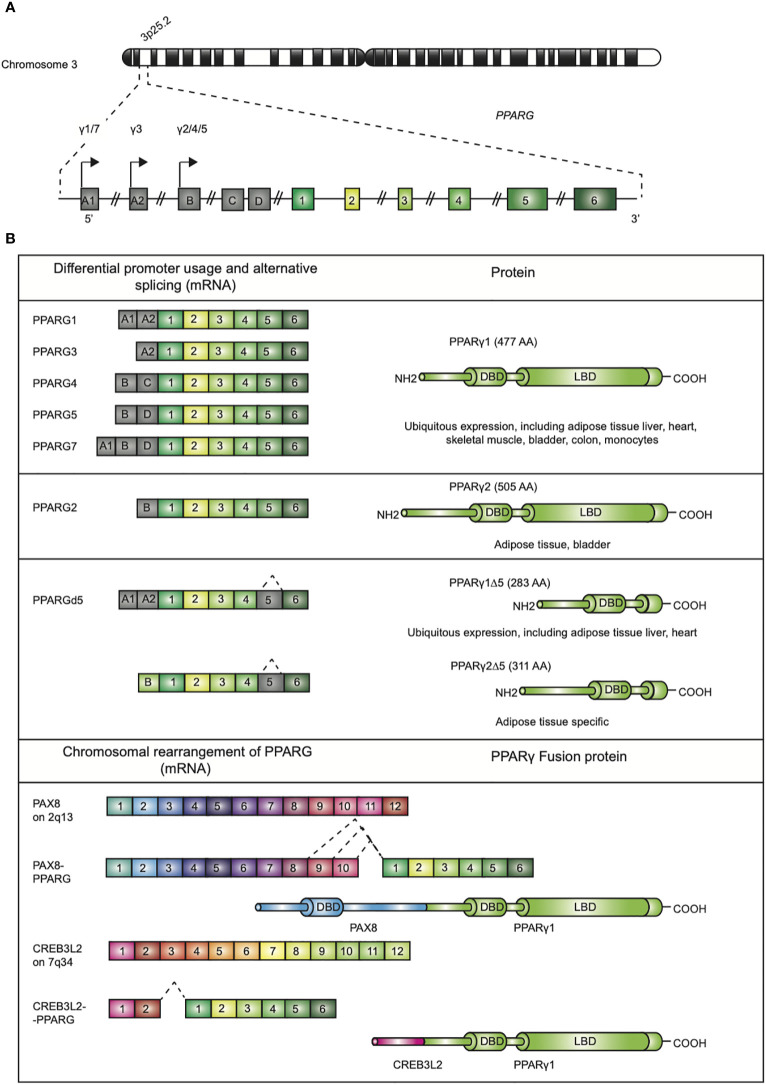
Genomic map of the PPARG gene on chromosome 3p25 and structure of PPARγ isoforms. **(A)** The gene *PPARG* is situated on chromosome 3p25. The gene encompassed 9 exons (exon A1-2, exon B-D, and exons 1-6). **(B)** Alternative promoter and mRNA splicing give rise to several PPARγ mRNA and protein isoforms. The mRNAs PPARG1, -3, and -4 translate into PPARγ1 (477 amino acids; AA). mRNA PPARG2 gives rise to PPARγ2 (505 AA). A third and fourth PPARγ protein isoform, denoted as PPARγ1Δ5 and PPARγ2Δ5, have been reported. These isoforms lack the ligand binding domain (LBD), which is due to alternative splicing. Chromosomal rearrangement of PPARγ leading to PAX8/PPARγ and CREB3L2/PPARγ fusion proteins, contains functional DBDs of both proteins, have been described in carcinogenesis.

Since its discovery in the early 1990s by Tontonoz et al (1)., the nuclear receptor PPARγ, encoded by the *PPARG* gene on chromosome 3p25.2 in humans (**Figure 1A**) (2), has been recognized as the master regulator of adipose tissue biology. The human *PPARG* gene, encompassing 9 exons, generates four PPARG splice variants (PPARG1-4) encoding for two protein isoforms *via* differential promoter usage and alternative splicing (**Figure 1B**) (3). The mRNAs PPARG1, PPARG3, and PPARG4 all give rise to the PPARγ1 isoform. PPARγ1 is a 477 amino acid protein that is broadly expressed with relative high levels in the adipose tissue, liver, colon, heart, various epithelial cell types, and skeletal muscle. In addition, PPARγ1 is expressed in numerous cells of the immune system, including monocytes/macrophages, dendritic cells, and T lymphocytes. The PPARG2 mRNA transcript translates into the PPARγ2 isoform. PPARγ2, containing an additional 28 amino acids in its NH2-terminus, is almost exclusively expressed in adipose tissue. This isoform is also expressed in urothelial cells (4, 5), which are highly specialized transitional epithelial cells that line the organs of the urinary system, including the bladder, and in regulatory T cells (Tregs) and other T cell populations, albeit that total PPARγ expression is low in non-Tregs (6). Recently, a third and fourth PPARγ protein isoform, denoted as PPARγ1Δ5, and PPARγ2Δ5, respectively, have been reported (**Figure 1B**) (7). PPARγ2Δ5 is endogenously expressed in adipose tissue and lacks the entire ligand binding domain (LBD) due to physiological exon 5 skipping (7). The endogenous expression PPARγΔ5 positively correlates with body mass index (BMI) in overweight or obese and type 2 diabetic patients. The naturally occurring PPARγΔ5 isoforms impair the adipogenic potential of adipocyte precursor cells by dominant-negative inhibition of PPARγ, which possibly contributes to adipose tissue dysfunction in obesity (7).

**Figure 2:**
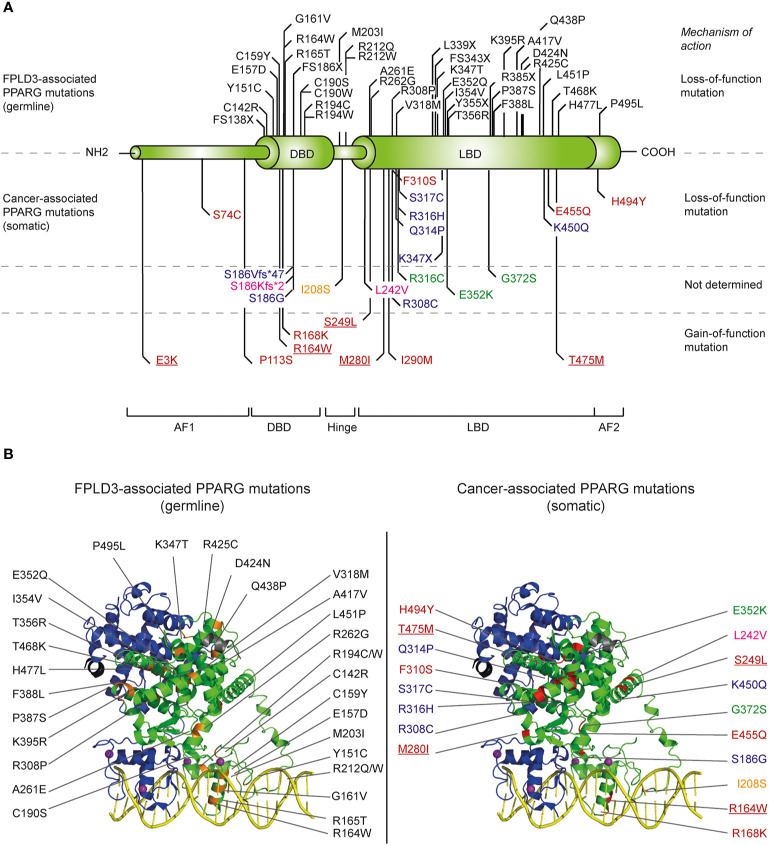
Overview of identified natural *PPARG* mutations implicated in FPLD3 and cancer. **(A)** Schematic representation of the distinct domains of PPARγ. Mutations indicated above the PPARγ structure are mutations are germline loss-of-function mutation, implicated in FPLD3. Mutations depicted below the PPARγ structure are somatic loss-of-function or gain-of-function mutations identified in different cancer types. Mutations have been identified in tissue form digestive tract (colon, stomach, oesophagus, and pancreas; indicated in blue), melanoma (green), breast cancer (pink), prostate cancer (yellow), and bladder cancer (red). Some bladder cancer-associated PPARγ mutations (underscored in figure) have also been identified in other types of cancer, including lung cancer (E3K), kidney cancer (R164W), endometrium cancer (S249L), melanoma (M280I), and diffuse glioma (T465M), respectively. **(B)** FPLD3 (orange, left panel) and cancer associated mutations (red, right panel) indicated in 3D representation, based on the crystal structure of PPARγ (green)-RXRα (blue) on DNA (yellow) with Rosiglitazone, 9-cis retinoic acid and NCOA2 peptide (grey) (PDB entry 3DZY).

PPARγ is a representative member of the nuclear receptor (NR) superfamily. To date, 48 NRs have been identified in human. NRs regulate various critical aspects in development, physiology, reproduction, and homeostasis. NRs are multi-domain ligand-inducible transcription factors that share a structural homology to a varying extent (8). Alike other NRs, PPARγ contains an autonomous transactivation domain 1 (AF-1) in the unstructured N-terminus (**Figure 2** **)**. The AF-1 domain is implicated in the constitutive ligand-independent activation of PPARγ target genes. Juxtaposed to the AF-1 domains is the DNA binding domain (DBD) that contains two zinc fingers required for DNA binding. The DBD connected to the ligand binding domain (LBD) *via* a flexible hinge region. In the case of PPARγ, this hinge region physically interacts with the DNA (9). The ligand binding domain (LBD) is situated in the C-terminus. The LBD is a complicated structure that is arranged in a conserved three-layered α-helical sandwich containing 12 α-helices and 4 β-strand elements (8). The LBD overlaps with the ligand-dependent transactivation domain 2 (AF-2). The LBD is a key domain for transactivation of PPARγ target genes as it is implicated in ligand binding, heterodimerization with binding partner retinoid X receptor alpha (RXRα), and interactions with transcriptional co-regulators.

**Figure 3:**
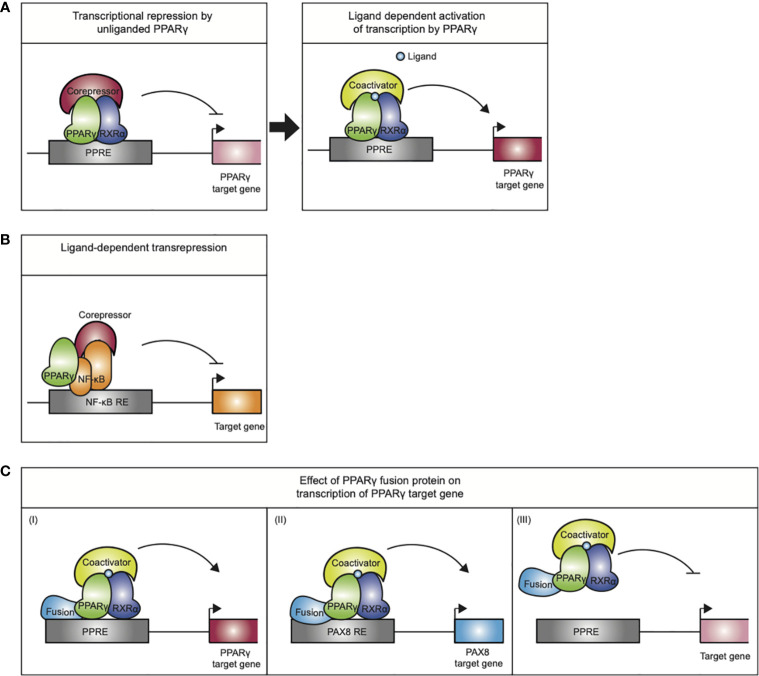
Mechanisms of action exerted by the PPARγ/RXRα heterodimer. **(A)** Transcriptional repression by unliganded PPARγ. Upon ligand binding the PPARγ/RXRα heterodimer undergoes a conformational change that promotes corepressor release and recruitment of coactivators, initiating transcription. **(B)** Ligand-dependent transrepression by antagonizing the NF-κB (and AP-1, not indicated) pro-inflammatory signaling pathways. This effect does not require DNA binding by the PPARγ. **(C)** The mode of action performed by PPARγ-fusion proteins in carcinogenesis is not completely understood. (I) altered expression of PPARγ target genes, (II) altered expression of PAX8 target genes (III) PPARγ fusion protein may act as a negative inhibitor of tumor suppression by inhibiting PPARγ target gene expression.

PPARγ exerts its gene regulatory potential *via* transactivation and transrepression (**Figure 3** **)**. Transactivation involves a mechanism by which PPARγ binds as a heterodimer complex with RXRα to PPAR response elements (PPREs) (10). PPREs consist of a hexameric repeat (AGGTCA) spaced by one or two nucleotides (referred to as DR1 and DR2 elements) (11), which are situated in promoter and enhancer regions of PPARγ target genes (12). Noteworthy, enhancers may not only loop to the nearest promoters, but can also increase transcription of their target genes *via* looping to promoters at greater genomic distances.

In the last decade, genome-wide binding profiles of PPARγ have been mapped in different cell types, including adipocytes and macrophages (13–17). These binding profiles have not only indicated that PPARγ binds to thousands of sites in the genome, of which many binding sites are located far from proximal promoters, but also that the PPARγ binding is highly context-dependent as binding sites differ between cell types and even between adipocytes from different anatomical locations (13–17). The context-dependency of PPARγ binding is at least in part mediated by cooperative binding to the chromatin with other adipogenic transcription factors, such as C/EBPα, followed by cooperative recruitment of coactivators (15).

Transcriptional control of the target genes by PPARγ furthermore depends on multiprotein coregulatory complexes that are recruited to the PPREs (18). In basal conditions, i.e., in absence of ligand, PPARγ/RXRα favors stable interactions with corepressor complexes, containing NCoR or SMRT, which recruit chromatin-modifying enzymes such as histone deacetylases that make the chromatin inaccessible to binding of transcription factors or resistant to their actions and thereby actively repress transcription (**Figure 3A**). Upon ligand binding, the PPARγ/RXRα heterodimer undergoes a conformational change that promotes corepressor release and recruitment of coactivators, like SRC1 and CBP. Coactivators enhance PPARγ transactivation by facilitating acetylation of the histone tails, making the chromatin less restrictive, and assembly of general transcriptional machinery. Next to the “classical” transactivation mechanism described above, PPARγ can also negatively regulate gene expression by a mechanism referred to as ligand-dependent transrepression (**Figure 3B**). This mechanism involves antagonizing the NF-κB and AP-1 pro-inflammatory signaling pathways, and has been mostly described in immune cells (19–23). In this case, PPARγ does not bind to DNA itself, and several studies indicate that PPARγ transrepresses genes as a monomer, i.e., independent of RXRα (23). While various mechanisms have been postulated for transrepression by different NRs (24–26), the most detailed mechanism proposed for PPARγ involves inhibition of co-repressor degradation. Pascual et al. (27) showed that clearance of NCoR/SMRT-HDAC3 complexes by proteosomal degradation from various AP1- and NFkB-regulated promoters (e.g., IL-8, Mmp12, and iNOS) upon activation is prevented in the presence of liganded, monomeric PPARγ.

Interestingly, the transrepression mechanism described above involves a specific post-translational modification, SUMOylation of lysine 365. In fact, to adequately processes external signals and adapt to relevant gene expression programs PPARγ activity is regulated by several, probably interconnected, post-translational modifications, including phosphorylation, acetylation, and the aforementioned SUMOylation [reviewed in (28)]. Depending on cellular context and the kinases involved, phosphorylation of PPARγ S112 can either impair or increase PPARγ activity (29). Phosphorylation of PPARγ S273 by Cdk5 does not affect its adipogenic capacity, but affects many PPARγ target genes that have been shown to be dysregulated in obesity (30). In addition, acetylation of K268 and K293 correlates with the phosphorylation status of S273 and favors lipid storage and cell proliferation (31). Selective adipocyte deletion of the deacetylase Sirt1 that deacetylates PPARγ K268 and K293 leads to dephosphorylation of S273 and improve metabolic functions (32).

Alike other NRs, PPARγ governs nutrient- and hormone-mediated responses. Despite intensive efforts, it is not clear whether PPARγ is *in vivo* activated by a specific, high-affinity, and endogenous ligand. PPARγ LBD crystal structures reveal a large ligand binding pocket (LBP), which not only allows for promiscuous binding of ligands with lower affinity, but also allows ligands to occupy the canonical LBP in different conformations (33). Indeed, the activity of PPARγ can be modulated by a variety of natural compounds, including polyunsaturated fatty acids (34), eicosanoids (35, 36), and oxidized lipid components (discussed below) (37), suggesting that PPARγ functions as a general lipid or nutrient sensor (34). However, the physiological relevance of these compounds is not exactly clear. Endogenous ligands not only bind with low affinity for PPARγ, also the physiological concentrations in mammalian cells are often insufficient to function as a physiological ligand (38). Alternatively, the physiological activation of PPARγ could be the resultant of combined effects of multiple ligands that simultaneously bind with different affinities to distinct subregions in the LBP (39), thereby inducing different PPARγ conformations with potential different biological outcomes (39).

PPARγ is the cognate receptor for thiazolidinediones (TZDs), a class of anti-hyperglycaemic drugs, including rosiglitazone and pioglitazone (40). TZDs stimulate adipogenesis (40) and cause a metabolically beneficial shift in lipid repartitioning from storage in visceral to subcutaneous adipose tissue depots as well as from ectopic storage in non-AT organs (e.g., liver muscle) to AT (41–43). TZDs and endogenous ligands have overlapping binding sites in the LBP, which potentially allows for binding competition to the same site. TZDs occupy the canonical LBP of PPARγ and by interacting with residues in helices 3, 5, 6, and 7 and the β-sheet, stabilizes the dynamics of helix 12 and the AF2 surface (44, 45).

Whereas TZDs are commonly referred to as full classical PPARγ agonists, TZDs have a separate biochemical activity: inhibition of the Cdk5-mediated phosphorylation of PPARγ at serine residue 273 (30). Phosphorylation of PPARγ S273 requires a physical interaction between CDK5 and PPARγ (46). The transcriptional corepressor NCoR is an adaptor protein for the physical interaction between CDK5 and PPARγ. Upon rosiglitazone the interaction between NCoR and PPARγ is reduced, which leads to i) derepression of PPARγ and activation of the PPARγ transcriptional program and ii) attenuation of the psychical interaction between CDK5 and PPARγ and subsequent reduced phosphorylation of S273 (46). Interestingly, MRL24 that displays poor agonistic activity but robust anti-diabetic activity in mice (47), was also very effective in inhibiting the Cdk5-mediated phosphorylation (30). This suggests that new classes of antidiabetic drugs that i) bind with high affinity to PPARγ, ii) specifically target the Cdk5-mediated phosphorylation of S273, and iii) completely lack the classical transcriptional agonism, hold promise for treatment of T2DM. The PPARγ ligand SR1664 was essentially displayed no transcriptional activity and was very effective in blocking the Cdk5-mediated phosphorylation (48). In obese mice, SR1664 displayed strong antidiabetic effects without adverse effects (48). However, unfavorable pharmacokinetic properties of SR1664 preclude its administration in human (48). Therefore, SR1664 should rather be considered as a proof-of-principle.

In addition to binding in the canonical LBP, a recent structure-function study shows that some PPARγ ligands denoted as noncanonical agonist ligands (NALs), like the aforementioned compound MRL24, and SR1664, can also bind to an alternate site of PPARγ (49). TZDs, including rosiglitazone and pioglitazone display less prominent alternate site functional effects (49). The alternate binding of PPARγ ligands can occur when the canonical LBP is occupied by the covalent antagonists or endogenous ligands. Although the exact mechanisms are not clear, alternate site binding stabilizes the AF2 surface, most likely indirectly *via* stabilization of helix 3. Furthermore, coregulator-binding assays indicate that alternate site binding has an impact on coregulator interactions, transactivation, and target gene expression (49). The identification of the alternate binding site has three important implications. Firstly, compounds that block phosphorylation of S273 with little transactivation might be complicated by alternate site binding if this site *in vivo* contributes to classical PPARγ agonism. Secondly, it needs to be defined whether some of the supposed PPARγ-independent effects of TZDs could in fact be mediated by the alternate site binding. Lastly, allosteric modulators that target the alternate site might be particularly relevant for obese individuals in which the probability that canonical LBP is occupied by oxidized fatty acids due to increased bioavailability of endogenous ligands is increased (49).

## PPARγ in Adipose Tissue

White, beige, and brown adipocytes have been identified in mammals. Although these three type of adipocytes rise from different precursors and differ significantly in their morphology and function, the cells all go through a well-orchestrated differentiation process to become mature and fully functional (50). During the various stages of the adipocyte lifespan, PPARγ is a well-established key player. Recently, a fourth type of adipocyte, denoted as pink adipocytes, has been described in in mammary glands of pregnant mice (51). During pregnancy, lactation, and post-lactation subcutaneous white adipocytes in murine mammary gland undergo a transdifferentiation process ending in milk-producing epithelial glandular cells that contain abundant cytoplasmic lipid droplets to meet the nutritional needs of the pups (51, 52). As the number of studies in pink adipocytes is limited so far, we will focus in this review on the role of PPARγ in white, brown, and beige adipocytes. In these cells, PPARγ exerts its essential functions primarily *via* “classical” transactivation of target genes.

### White Adipocytes

White adipose tissue (WAT) is the most abundant adipose tissue in the human body (53). Mature white adipocytes are unilocular cells composed of a large lipid droplet occupying ~95% of the cellular volume. Depending on the size of the lipid droplet, the cell size varies from 20 to 200 µM (54). The *in vivo* regulation of adipocyte development, including the stem cell commitment toward white adipocytes, is poorly understood. Adipocyte-lineage tracing, which so far can only be performed in mice, indicate that white adipocytes can be derived from both Myf5^−^ and Myf5^+^ precursor cells (55). The Myf5-lineage distribution in adipose tissue is dynamic and can be affected by ageing and diet. The Myf5^−^ and Myf5^+^ white adipocytes can compensate for each other during development, reflecting adipose tissue plasticity (55). In mice, depot-dependent variations were observed among the degree of plasticity (55). Although it remains to be defined whether this concept also applies to human adipocytes, a heterogeneity in adipocyte origins may explain the heterogeneity in adipose tissue depot function and contribute to adipose tissue patterning variations in the human population (55). After stem cell commitment toward white adipocyte lineage, the expression and activation of PPARγ is both sufficient and crucial to initiate the adipogenic differentiation program and maintain adipocyte phenotype, integrity, and function, based on a large set of different genetic mouse models (56). PPARγ primarily regulates the expression of genes implicated in adipocyte differentiation and adipocyte maintenance. In addition, PPARγ governs the expression genes involved in various processes in lipid and glucose metabolism including lipogenesis (e.g., *LPL, ANGTPL4*, and *CIDEC*), fatty acid transport (e.g., *FABP4*), and gluconeogenesis (e.g., *PEPCK, GYK*, and *AQP7*).

The importance of PPARγ for white adipose tissue biology in humans is underscored in patients suffering from familial partial lipodystrophy subtype 3 (FPLD3), a rare autosomal dominant inherited condition caused by loss-of-function mutations in the *PPARG* gene [reviewed in (28)]. Patients with FPLD3 lack subcutaneous adipose tissue in the extremities and gluteal region combined with lipohypertrophy in the face, neck, and trunk, and suffer from multiple metabolic complications including type 2 diabetes mellitus (T2DM). Since the first report of a germline loss-of-function mutation in *PPARG* in patients with FPLD3 (57) an increasing number of FPLD3-associated mutations in *PPARG* has been identified [reviewed in (28)]. The FPLD3-associated PPARγ mutations are mainly situated in either the DBD or LBD (**Figure 2**). Mutations in the DBD interfere in efficient DNA binding. Mutations affecting the LBD—which are scattered over the whole LBD, based on crystal structures (**Figure 2**)—often cause multiple molecular defects by impairing heterodimerization with RXRα, ligand- and/or cofactor binding (18).

Taken together, genetic mouse models together with the FPLD3-associated PPARγ mutations indicate that PPARγ plays a key role in white AT differentiation, function, and maintenance. The dominant mode of action in this biological setting appears to be “classical” transactivation: the majority of genes regulated by PPARγ in white adipocytes rely on direct DNA binding, and FPLD3-associated PPARγ mutations do not alter transrepression, although this is not studied frequently (58).

### Brown Adipocytes

Brown adipose tissue (BAT) emerged approximately 150 million years ago in mammals (59). BAT is unique for endothermic placental mammals and makes it possible to maintain a body temperature that is higher than the ambient temperature by producing heat independently of shivering and locomotor activity. This process is also referred to as non-shivering thermogenesis (59). BAT is richly innervated and vascularized and is composed of brown adipocytes (~40 µM in size) that contain multilocular lipid droplets and a large number of mitochondria (54). BAT derives its brown color from the conspicuous iron-rich mitochondrial mass. BAT uniquely expresses the gene *UCP1*, which encodes for uncoupling protein 1 (UCP1), located in the inner mitochondrial membrane. When activated, UCP1 mediates non-shivering thermogenesis by uncoupling of the oxidative phosphorylation from ATP synthesis, thereby provoking 1) dissipation of chemical energy in the form of heat and 2) stimulating high levels of fatty acid oxidation (60).

BAT is present in dedicated depots. In rodents, BAT is abundantly present throughout life. In human adults, BAT is located mainly cervical/axillary, perirenal/adrenal, and in the mediastinum along large blood vessels, trachea, and surrounding the intercostal arteries (59). In new-born infants, BAT is also situated between the shoulder blades as a thin kite-shaped layer (60). Although BAT depots regress with increasing age and can become even indistinguishable from WAT, healthy adults retain metabolically active BAT (61–63). For instance, positron emission tomography (PET) and computer tomography (CT) in human indicated that BAT-mediated thermogenesis is activated and increases in size by cold exposure (61–63). This process is also known as BAT recruitment. Depending on the size of the BAT depots, thermogenesis can account for up to approximately 15% of the total daily energy expenditure (64). Therefore, increasing energy expenditure by activation of BAT has been suggested as a therapeutic strategy for treating obesity (65).

Mice studies indicate that PPARγ functions is a master regulator in BAT (66). BAT-specific PPARγ knock out mice showed reduced wet weight of BAT, smaller brown adipocytes, and smaller lipid droplets when compare to wild type animals. However, there was no difference in total body weight or body composition (67). Furthermore, it was also shown that loss of PPARγ inhibited the ability of brown adipocytes to respond to β -adrenergic stimulus in *in vitro* cultures (67). An increase in non-shivering thermogenesis was observed in mice treated with TZDs (68, 69), and *in vitro* studies showed that activation of PPARγ in brown adipocytes leads to increase in adipogenesis and increase in lipid metabolism (70). Additional studies pointed at PPARγ as crucial regulator of UCP1 expression and BAT function (71). Specific BAT PPARγ target genes have been described (FABP3 and GYK), and particularly the de-acetylation of K268 and K293 of PPARγ by SIRT1 have been linked to BAT (32). De-acetylation of these residues is required for the recruitment of Prdm16, an essential cofactor in BAT (72). Moreover PGC1a, one of the most well-known regulators of BAT, has also been identified as a cofactor of PPARγ in BAT (73).

Collectively, PPARγ plays a key role in BAT differentiation and function, which most likely relies on “classical” transactivation, although transrepression cannot be excluded given the limited number of studies. BAT-specific molecular mechanisms, which may be different from WAT, could involve for example specific transcriptional cofactors (73), but details remain to be fully elucidated.

### Beige Adipocytes

Mammals possess a second type of thermogenic adipocytes: beige adipocytes, also denoted as “brite” (brown-like in white) adipocytes (74). Beige adipocytes are inducible thermogenic cells that are sporadically located in white adipose tissue depots (74). Beige adipocytes share many morphological and biochemical features with brown adipocytes (**Figure 1**) (60). Alike brown adipocytes, beige adipocytes contain multiple small lipid droplets and a large number of mitochondria that express UCP1. Recruitment of beige adipocytes, referred to as “browning” or “beigeing/beiging” of white adipose tissue, is induced in response to environmental conditions, including chronic cold exposure, exercise, long-term treatment with PPARγ agonists or β3-adrenergic receptor agonists, cancer cachexia, and tissue injury (75). It is currently unknown whether beige adipocytes arise through transdifferentiation from pre-existing white adipocytes or by *de novo* adipogenesis from a precursor cell pool, or both (76).

Although, the exact mechanism by which PPARγ agonists induce browning of white adipocytes is not exactly known, PPARγ agonist require full agonism to activate the browning fat program. The effect is at least in part mediated by PRDM16, a factor that as described above is essential in the development of classical brown fat (77). Therefore, it is likely that in beige adipocytes, alike brown adipocytes, “classical” transactivation by PPARγ is an important mechanism of action.

## PPARγ in Immune Cells

Even though PPARγ is the master regulator of adipocyte differentiation and function (78), already in one of the first publications showed high PPARγ expression in mouse spleen (79) suggesting a role for PPARγ in immune cells. In fact, PPARγ is expressed in a variety of immune cells and its role and importance have been investigated during the last twenty years (80–82). Although PPARγ expression have been described in several types of immune cells we will focus on monocyte/macrophages and dendritic cells as part of the innate immune system, and T cells of the adaptative immune system.

As described above for adipocytes, PPARγ plays a role in determining the cellular phenotype by regulating differentiation (adipogenesis) and function (e.g., lipid metabolism and secretome) by directly activating the transcription of so-called PPARγ target genes. Similar molecular mechanisms are in place in immune cells, and also here PPARγ can deterimine cellular phenotype: amongst others, PPARγ 1) regulates macrophage differentiation, 2) regulates classical/alternative macrophage activation (“polarization”), 3) controls lipid metabolism in multiple immune cell types, and 4) plays an immune-modulatory role. PPARγ function in immune cells could also be categorized according to its mechanism of action, with the regulation of lipid metabolism and the ability to induce differentiation of immune cells more linked to “classical” transactivation, while the transrepression activity of PPARγ is more important in its immunomodulatory role and both mechanisms are involved in macrophage activation.

### Transactivation by PPARγ in Immune Cells

PPARγ can directly activate the transcription of target genes in immune cells through direct DNA binding, similar to its activity in adipocytes described above. As mentioned earlier, the genomic locations where PPARγ binds and the target genes partly overlap between, for example, adipocytes and macrophages, but cell-type specific regulation may depend on cooperation with other transcription factors like PU.1 and STAT6 (17, 83).

PPARγ expression is highly induced during monocyte to macrophage differentiation (84–86), and although initial studies using embryonic stem cells suggested that PPARγ is dispensable in this process (87), more recent studies have demonstrated that PPARγ is essential for the differentiation of fetal monocytes into alveolar macrophages (88). In mature macrophages, PPARγ was found to cooperate with *PU1* specifically on monocyte-unique target genes (17), reminiscent of the interplay between PPARγ and C/EBPa in adipocytes mentioned earlier. PPARγ is also expressed in several dendritic cell (DC) subtypes and is also highly upregulated in monocyte-derived DC differentiation (89, 90). Although the importance of PPARγ in immune cell differentiation is evident, little is known about the exact function of the receptor in these differentiation processes. Better models are required as well as studying the contribution of PPARγ in a more cell-type specific way.

Next to macrophage differentiation, PPARγ is also an important regulator in macrophage polarization, where PPARγ activation drives the alternative M2 macrophage phenotype (91–93). Alternatively activated macrophages (M2 phenotype) can be induced by IL-4, IL-10, and IL-13 and are characterized by the expression of several genes including Arg1 and Mgl1/CD301a, CD-204 and mannose receptor/CD163, and IL-10 and transforming growth factor beta (TGF-β). Some of these, including Arg1 and Mgl1 (94), are direct PPARγ target genes. Furthermore, PPARγ expression is induced by IL-4/STAT6 signaling as well as IL-13 (95), and STAT6 functions as a “facilitator” of PPARγ signaling, all supporting the idea that PPARγ is crucial for the anti-inflammatory M2 phenotype in macrophages. It was recently found that PPARγ contributes to maintain a chromatin structure that facilitates the binding of STAT6 and polymerase II upon repeated IL-4 treatments. PPARγ recruits the coactivator P300 and RAD21 to the DNA and thus reinforcing a M2-like phenotype in macrophages (96), is worth mention that this function of PPARγ is independent of ligand binding.

Next to macrophage and DC differentiation and macrophage polarization, PPARγ can also directly regulate lipid metabolism in immune cells (37, 87, 92, 97, 98), reminiscent of its role in white and brown adipocytes. In monocytes, macrophages, and dendritic cells, PPARγ directly regulates the expression of genes involve in lipid transport and metabolism such as the class B scavenger receptor *CD36* (99)*, FABP4, LXRA, and PGAR* (86). The use of PPARγ ligands in these cells has shown that the expression of these genes is upregulated upon treatment and downregulated when treated with PPARγ antagonists (100). The CD36 protein is also involved in macrophage uptake of oxLDL, but at the same time PPARγ directly activates an LXR-ABCA1 pathway for cholesterol efflux (97). In DCs PPARγ also plays a key role in lipid homeostasis by directly regulating many “known suspects” (101) but it also regulates another aspect of lipid homeostasis and lipid antigen presentation. Activation of PPARγ gives higher expression of CD1d, a molecule involved in the presentation of lipid antigens to T cells, resulting in a DC subtype with increased potential to activate iNKT cells (100, 102, 103). These findings indicate that PPARγ has a functional role in the modulation of the immune response through DCs beyond regulation of more classical lipid metabolism pathways.

Changes in the lipid microenvironment can trigger different DC functions that regulate the immune response (104). PPARγ classical transactivation role bridges the lipid microenvironment and the DC function by activating genes involve in lipid transport, metabolism, and presentation.

The classical role of PPARγ as a gene activator has also been studied in T cells and again relates to lipid metabolism (81, 82). T cells can be subdivided into cytotoxic T cells, T helper, and regulatory T cells (Treg), and the T helper cells can be further classified depending on the phenotype into Th1, Th2, and Th17; less well characterized are Th9 and Th22 subsets. Regardless of the subtype of T cell, activation of PPARγ is linked to an activation of genes related to lipid metabolism (CD36 and FABPs) indicating the importance of PPARγ in this process. Special mention deserves the visceral adipose tissue resident regulatory T cells (VAT Tregs), in which PPARγ has been implicated in its function and development (6). VAT Tregs represents a unique subtype of cells in which the expression of PPARγ positively correlates with the expression of chemokines and chemokines receptors (Ccr2, Cxcl3, and Cxcr6) that regulates leukocyte migration and infiltration, lipid metabolism genes, and IL10. Interestingly, the PPARγ1 and PPARγ2 isoforms induce the same genes upon activation in VAT Tregs (mainly related to lipid metabolism) but differ in the genes that they downregulate (6), the latter happening most likely through the mechanism of transrepression.

### Transrepression by PPARy in Immune Cells

The role of PPARγ as an immune-modulator, and in particular a repressor of inflammation, has been studied in most detail in macrophages and T cells (19–22, 93). Although the transrepression activity of PPARγ is probably not exclusive to immune cells, this immunomodulatory role is a good example of the importance of this specific mechanism of action of PPARγ.

In macrophages it has been shown that activation of PPARγ using TZDs suppresses the production of pro-inflammatory cytokine, such as TNFα, IL-1B, and IL-6 (19, 93) and the expression of other genes involved in inflammation, including iNOS and MMP9, in a dose-dependent manner. As described above, inhibition of the transcription factors NFkB and AP-1 is the most widely studied mechanism, but other mechanisms are also possible (23). Similarly, in DCs PPARγ ligands downregulate chemokines and receptors (IL-12, CD80, CXCL10, RANTES) that recruit Th1 lymphocytes (100, 102). In addition, PPARγ activation in DC may impair the migration of these cells to the lymph nodes, and this might be partially due to inhibition of CCR7 by PPARγ (102, 105).

The role of transrepression by PPARγ in T cells has been the object of intensive discussion during the last two decades (81, 82, 106), as this mechanism of action was implicated in seemingly conflicting biological processes. Initial studies suggested that PPARγ had an inhibitory effect on T cell proliferation (107), and that the underlying mechanism involved transrepression of the IL2 gene: activated PPARγ was shown to bind to nuclear factor of activated T cells (NFAT) and repress its activity and binding to the IL-2 promotor (107, 108). Besides T cell proliferation, PPARy-mediated transrepression was reported as a repressor of excessive Th1 response, by on the one hand inhibiting production of the Th1 cytokine and antigen-specific proliferation and on the other hand controlling Th2 sensitivity to IL-33 (109, 110). In fact, Cunard and colleagues showed that PPARγ binds to the IFNγ promoter and is able to repress its expression when T cell were treated with PPARγ ligands, and that IFNγ expression was enhanced when cells were treated with PPARγ antagonist GW9662 (111). The underlying mechanism was proposed to be inhibition of AP-1 activity, similar to the transrepression mechanism in macrophages. However, while these studies suggest a pro-Th2 role for PPARy mediated transrepression, PPARγ was also reported to be involved in the downregulation of well-known Th2 cytokines like IL-4, IL-5, and IL-13, again through interaction with NFAT (112). Altogether, these studies indicate that the role of PPARγ in the modulation of the Th2 response in T cells remains unclear and further research is needed to fully elucidate its function. Finally, PPARy-mediated repression is important for Th17 differentiation, as lack of PPARγ leads to increased Th17 differentiation while activation of PPARγ was shown to have inhibitory effects (22). PPARγ recruits NCoR and SMRT to the *Rorc* promoter, thereby inhibiting IL-17a expression, and blocks IL-6 signaling by inhibiting the DNA binding activity of STAT3 (20, 21).

In summary, transrepression by PPARγ—where it counteracts other transcription factors like NFkB, AP-1, NFAT, and STAT3—may be a major molecular mechanism that drives the functional phenotype(s) and secretory output of macrophages, dendritic cells, and T cells. Findings in T cells appear sometimes conflicting, which makes it difficult to assign a clear pro-Th1 or pro-Th2 role to PPARy activation. It also indicates that the use of ligands in these cells might “hide” some of the PPARγ functions and more subtle approaches, such as the use of cels harboring specific PPARγ mutations or selective PPARγ modulators, must be used in order to fully elucidated PPARγ role in immune cells, taking the complex interactions between immune cell population into account.

## PPARγ in Cancer

Cancer is driven by the acquisition of genome instability. The cancer genome landscape contains an enormous diverse repertoire of amplifications, deletions, inversions, translocations, point mutations, loss of heterozygosity, and epigenetic changes that collectively result in tumorigenesis. The role of PPARγ in tumorigenesis is controversial. A large body of evidence suggests that PPARγ functions as a tumor suppressor, as activation of the PPARγ/RXRα signaling pathway in different types of cancer, including colon (113), lung (114, 115), pancreatic (116), prostate (117), and breast (118, 119) cancers, leads to inhibition of cell growth, decreased tumor invasiveness, and reduced production of proinflammatory cytokines. In addition, treatment with TZDs was shown to increase sensitivity to chemotherapy through downregulation of Metallothionein genes (120) and/or endotrophin (121), which may be linked to ligand-mediated prevention of S273 phosphorylation (122).

Furthermore, in lung cancer cells, a tumor suppressive function of PPARγ was contributed metabolic reprogramming (123), an essential biochemical adaptation required for cancer viability that is considered to be a crucial emerging hallmark of cancer (124). In contrast, a protumorigenic role for PPARγ has been suggested in a variety of cancers as well (5, 125, 126). Here, we will discuss several loss-of-function and gain-of-function mechanisms by which PPARγ can be implicated in tumor initiation and progression in several major cancers. In addition, we will address the yet partly undefined role of PPARγ fusion proteins in cancer.

### Transactivation by PPARγ

#### Loss-of-function Mutations

As discussed above, the PPARγ1 isoform is highly expressed in colon epithelial cells. The role of PPARγ in the development of normal colon epithelium and colorectal cancers is not completely understood and seems to be dual. The growth and differentiation of many colorectal cancers can be considerably inhibited upon ligand activation of PPARγ1 (113). This finding suggests that PPARγ functions as a tumor suppressor during colorectal carcinogenesis. In line with this, somatic *PPARG* mutations have been reported in ~8% of sporadic colorectal cancers (**Figure 2**). Genetic and epigenetic phenomena due to genetic alterations in other genes, like *RAS*, can further decrease PPARγ function in colon cancer. Activating mutations in *RAS* for example can result in hyperactivation of ERK1/2 and JNK pathways and ultimately impair PPARγ activity (28). Whereas all FPLD3-associated *PPARG* mutations that have been reported to date lead to mutant proteins that show a consistent and profound impairment in the transcriptional activity of PPARγ, the functional effects of colon cancer-associated *PPARG* mutations vary considerably (127). So far, six unique somatic *PPARG* mutations in colorectal cancers have been reported (128, 129). A side-by-side analysis of these colon-cancer associated mutants with some FPLD3-associated PPARγ mutants, shows that the colon-cancer associated mutants do not consistently display profound intra- and/or intermolecular defects (127). Moreover, while the abovementioned studies suggest that PPARγ functions as a tumor suppressor during colorectal carcinogenesis, it should be noted that other studies suggest that PPARγ activation increases the risk of developing colorectal cancer. Ligand-activation of PPARγ in *min* mice, an animal model for familial adenomatous polyposis due to mutations in the *APC* gene, results in a considerably greater number of polyps in the colon (125). Follow-up studies are clearly needed to reconcile these apparently conflicting findings and assign a clear role to PPARy in colon cancer.

In basal bladder tumors, four non-recurrent loss-of-function PPARγ mutations (S74C, F310S, E455Q, and H494Y, **Figure 2**) have been identified (130). All four PPARγ mutants display significantly reduced transcriptional activities. Biochemical and biophysical analysis of amino acid residues F310 and H494, situated in helix 3 and 12, respectively, indicated that both residues are essential for proper stabilization of helix 12. F310S and H494Y favor an inactive conformation, impairing both a proper release of corepressors and recruitment of coactivators (130). Basal tumors rely on EGFR signaling for growth (131). Interestingly, in basal cell lines the overexpression of wildtype but not H494Y, downregulates EGFR signaling.

Although the cancer-related PPARγ mutants—which are mainly scattered throughout the LBD (**Figure 2**)—may display variable and more subtle, i.e., context-dependent, intra- and/or intermolecular defects than the FPLD3-associated PPARγ, the cancer-related PPARγ mutants (**Figure 2**) are impaired in their ability to exert “classical” transactivation.

#### Gain-of-Function Mutations

In addition to its well-established role as master regulator in adipocyte biology, PPARγ has also been shown to be involved in the terminal differentiation of urothelium (4), a layer of specialized epithelial cells lining the lower urinary tract. However, little is known about its function in the bladder and in the pathogenesis of bladder cancer. In 12–17% of the muscle-invasive bladder carcinomas (MIBC) and in 10% of the non-muscle-invasive bladder carcinomas, *PPARγ* focal amplifications leading to PPARγ overexpression have been reported, suggesting a role for PPARγ in the initiation and maintenance of bladder cancer. MIBC are biologically heterogeneous and can further be grouped into basal and luminal subtypes (132). PPARγ has a protumorigenic role in luminal MIBCS, as the loss of PPARγ expression impairs the bladder cancer cell viability (133). These luminal tumors maintain molecular urothelial differentiation, even in the loss of morphological differentiation (133). This molecular differentiation depends on PPARγ (133).

In approximately 5% of the MIBCs and the luminal subgroup of MIBCs hotspot mutations of RXRα (S427F/Y) has been identified. These RXRα mutations rely on the introduction of an aromatic amino acid residue that enhances the ligand-independent activation of PPARγ (134). Tumors harboring RXR S427F/Y display enhanced expression of genes implicated in adipogenesis and lipid metabolism, including *ACOX1, ACSL1, ACSL5*, and *FABP4* (135). In addition, the RXRα hotspot mutations stimulate the proliferation of urothelial organoids, render bladder tumor cell growth PPARγ-dependent, and favor tumor evasion by the immune system.

Recently, seven recurrent driver gain-of-function PPARγ mutations have been identified in luminal bladder tumors (E3K, S249L, M280I, K164W, and T475M) (5). The mutations occur throughout the protein, affecting the N-terminus, DNA-binding domain, and ligand-binding domain (**Figure 2**). One recurrent mutation (E3K) was specific to the PPARγ isoform as it was situated in the N-terminal end. Functional analysis indicates that five mutations promote the transcriptional activity of PPARγ, which renders PPARγ-dependence to the cells. The three recurrent LBD-mutations promote, in absence of PPARγ ligands, the adoption of the active conformation of PPARγ by stabilizing helix 12 and induce recruitment of co-activators. Interestingly, four of the seven recurrent PPARγ mutations have also been identified in other types of cancer, including lung cancer, kidney cancer, cutaneous melanoma, and diffuse glioma (**Figure 2**) (5). Furthermore, other recurrent mutations that have not been identified in bladder cancer, have been identified in other types of cancer, including melanoma and prostate cancer (**Figure 2**) (5). Surprisingly, one of these recurrent PPARγ mutations, which are yet functionally uncharacterized, results in the same amino acid changes as FPLD3-associated loss-of-function PPARγ mutations (e.g., R164W and E352Q/K). This may indicate that a potential loss-of-function or gain-of-function effect is context dependent.

Although, not all recently identified gain-of-function PPARγ mutants have extensively been characterized and even affect different domains in the protein, at least some of the mutants have implications for “classical” transactivation of PPARγ target genes in bladder cancer.

### Somatic PPARγ Fusion Proteins in Cancer

Besides the loss- and gain-of-function mechanisms described above, a third way in which PPARy may be involved in carcinogenesis is represented by PPARG gene fusions observed in follicular thyroid carcinomas (FTCs). The t(2;3)(q13;p25) chromosomal translocation results in a *PAX8/PPARG* fusion gene that is detected in approximately 35% of FTCs and in a subset of follicular variant of papillary thyroid carcinomas (136). This chromosomal rearrangement is occasionally present in follicular adenomas as well (137). The gene paired-box gene 8 (*PAX8*) encodes for a member of the paired box (PAX) family of transcription factors and is a critical regulator in physiological thyroid development (138). In addition, PAX8 promotes the thyroid progenitor survival en in the mature thyroid it drives the expression thyroid specific genes, including genes encoding for thyroglobin and thyroid peroxidase (138, 139). The endogenous expression of *PPARG* in the thyroid is extremely low and it remains to be defined whether PPARγ has a physiological function in the thyroid (140). The translocation t(2;3)(q13;p25) results in a fusion transcript, driven by the *PAX8* promoter, wherein most of the coding sequence of PAX8 is fused in-frame to the entire coding sequence of PPARγ1 (141). The PAX8-PPARγ fusion protein (PPFP) contains functional DBDs of both the PAX8 and PPARγ (142). *In vitro* and *in vivo* evidence indicates that the PAX8-PPARγ fusion protein can function as an oncoprotein i) by acting as a negative inhibitor of tumor suppressor PPARγ or as ii) a novel transcriptional factor with proto-oncogene activity. Nevertheless, the expression of *PAX8-PPARG* in FTCs does not affect prognosis (143).

A second chromosomal translocation, t(3;7)(p25;q34) resulting in a *CREB3L2/PPARG* fusion gene, is a low incidence fusion mutation that is found in <3% of the FTCs (144). The gene cAMP Responsive Element Binding Protein 3 Like 2 (*CREB1L2*) encodes for a member of the bZIP transcription factor family. The CREB3L2/PPARγ fusion protein consists of amino acids 1 to 106 of wildtype CREB3L2, a new glutamic acid at position 107 juxtaposed to the all 477 amino acids of wildtype PPARγ1 (144). The CREB3L2/PPARγ fusion protein stimulates cell growth of transduced primary thyroid cells by inducing proliferation (144). The fusion protein seems to be unresponsive to thiazolidinediones. In addition, CREB3L2/PPARγ interferes in the CRE-related transcription as overexpression of CREB3L2/PPARγ inhibits the transcription of native cAMP-responsive genes in normal thyroid cells (144). The impaired ability to stimulate transcription is consistent with the loss of CREB3L2 bZIP domain, implicated in dimerization and DNA binding, in the CREB3L2/PPARγ fusion protein (144). The oncogenic activities of the CREB3L2/PPARγ fusion protein are most likely (at least in part) due to 1) disruption one functional CREB3L2 allele and 2) inhibition of cAMP responsive genes by interfering in CREB3L2 DNA-binding (144).

Taken together, the PPARγ fusion proteins display a third mode of PPARγ action, as they potentially alter the target gene profile of both parent proteins in the chimeric protein (**Figure 3C**) and will target multiple signaling pathways implicated in cancer.

Since the identification of the *PPARG* gene in the early 1990s the role of PPARγ in cancer has extensively been studied in many different human cancer cells and animal models. However, the biological significance of PPARγ in cancer development and progression is far from completely understood and for some cancers appears to be even inconsistent and contradicting. At best, the overall conclusion from these studies is that the context, e.g., specific tumor type, tumor stage, and tumor microenvironment, determines the exact role and function of PPARγ in human cancer. Therefore, cell-culture studies are limited in representing the complex gene-gene and gene-environment molecular interactions that are implicated in cancer onset and progression.

## Future Perspectives

**Figure 4:**
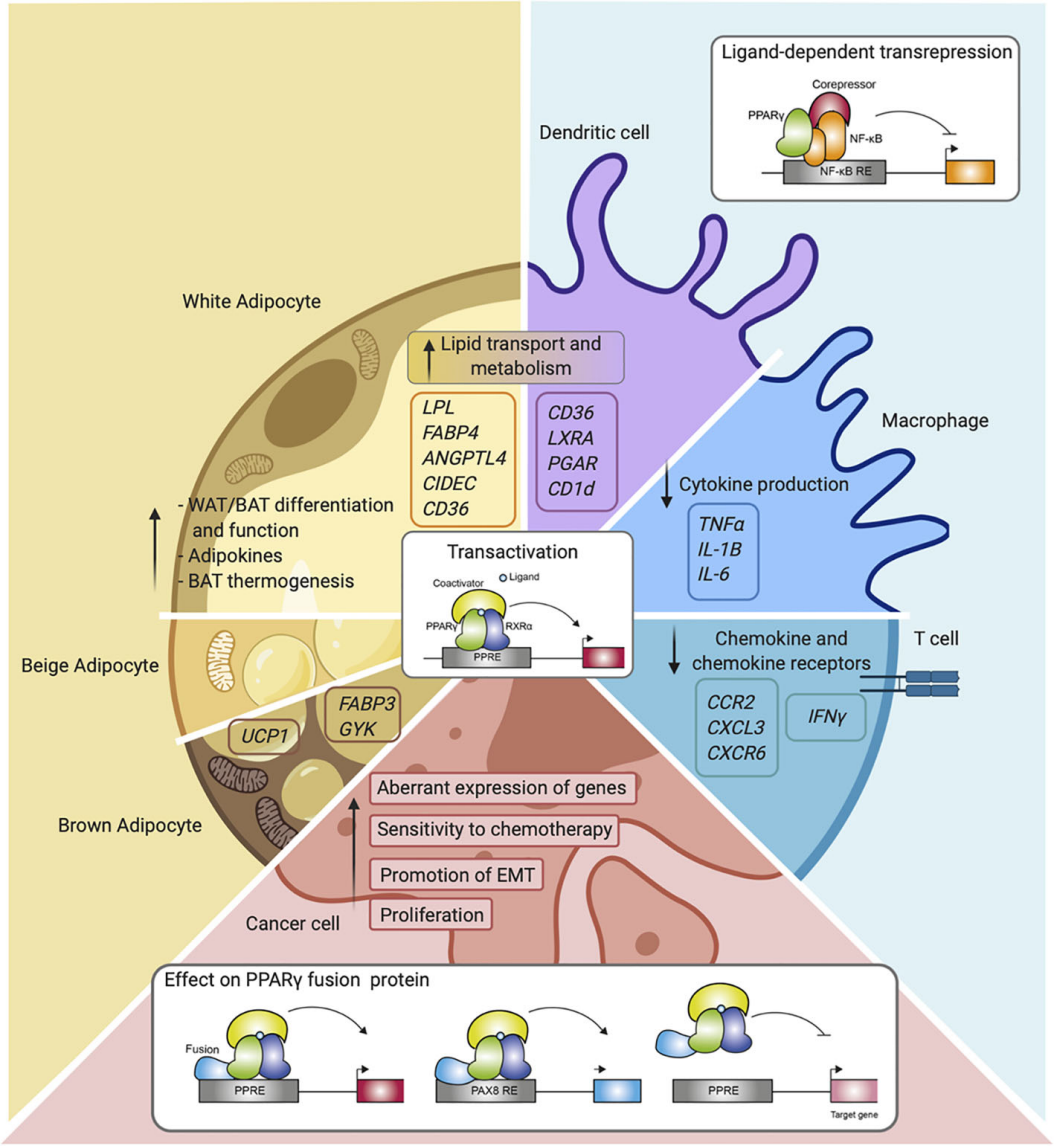
Overview of PPARγ function and mechanisms in the different cell types.** **Schematic representation of PPARγ in adipocytes (white, beige, and brown adipocytes), immune cells (macrophages, dendritic cells, and T cells), and cancer cells. Indicated are different cellular processes and mechanisms of action in which PPARγ is involved.

For many years, PPARγ was referred to mainly as the master regulator of adipocyte function, and although its expression in the immune system was already described in early research, its actual role in these cells only became apparent later (**Figure 4**). Nowadays, the immunomodulatory role of PPARγ in several immune cells is well-established as described in this review. While PPARy clearly functions in gene transactivation in both adipocytes and immune cells, gene repression by PPARy has been predominantly investigated in immune cells. PPARγ has also emerged as a factor involved in cancer onset and progression of several cancer types in recent years. Also, in this case, transactivation mechanisms are clearly relevant, underscored by both loss-of-function and gain-of-function mutations. It should be noted however that no single unifying role for PPARy in human cancer emerges, and that transrepression has not always been studied specifically. Finally, gene fusions with other gene products (PAX8, CREB3L2) as reported in specific carcinoma presents a third way in which PPARy regulates gene expression, resulting in either altered target gene sets and/or loss of activation.

It is well known that PPARy is the molecular target for TZDs, these drugs have been widely used for the treatment of hyperglycemia and T2DM. TZDs stimulate the expression of genes implicated in lipid uptake and storage (145) and consequently the levels of ectopically stored and circulating lipids are decreased. In addition, TZDs also increase the expression of adiponectin, which contributes to enhance insulin sensitivity of the liver, and improves hepatic steatosis (145). Given its central role in adipocyte biology and energy homeostasis, there is a clear rationale behind therapeutically targeting PPARy and improving insulin sensitivity. However, the use of TZDs is curtailed due to serious side-effects [review in (146)]. Although some side-effects, such as troglitazone-associated hepatotoxicity and rosiglitazone-associated myocardial infarction have been solved (147), others are still present. These common side-effects include weight gain, fluid retention, and osteoporosis. These unwanted side-effects are due to the ubiquitous expression of PPARy1 in combination with the full agonism characteristics of TZDs. As indicated earlier, new generations of ligands, referred to as noncanonical agonist ligands (NALs) and selective PPARy modulators (SPPARMs), hold promise in that respect. In fact, very recently, it has been shown how selective modulators of PPARy can improve liver histology without affecting body weight in biopsy-confirmed mouse model of nonalcoholic steatohepatitis (NASH) (148).

Similar to being a potential drug target in metabolism, PPARy could represent a therapeutic target for a variety of cancers because of its ability to be selectively activated through its LBD. As indicated above, various parameters including tumor type and genetic background must be taken into account, as PPARy displays oncogenic and tumor suppressor roles. Nonetheless, targeting PPARγ in the cancer context can be effective. In pancreatic ductal adenocarcinoma for example, the fourth most frequent cause in cancer-related deaths, PPARγ ligands have shown promising results *in vitro* and *in vivo* increasing apoptosis and reducing tumor growth, respectively (149, 150).

While we have described above that PPARy is expressed in multiple cancer cell types, and PPARy ligands can affect cancer cell function and behavior (e.g., proliferation and sensitivity to chemotherapy), some of the anti-cancer effects may actually occur indirectly through adipocytes surrounding the tumor or distal adipose tissue. PPARγ plays a crucial role in AT, and as it has been shown before, AT influences cancer initiation and progression through several mechanisms (151). It is estimated that obesity contributes to up to 20% of cancer-related deaths. Obesity is associated with increased risk of cancer development (i.e., colorectal, post-menopausal breast, and kidney among others) but the association with poor prognosis is even stronger for some of these cancer types. Obese AT is characterized by a chronic low-grade inflammation that leads to dysfunctional adipocytes, metabolic dysregulation, and secretion of pro-inflammatory cytokines are some of the factors that have been correlated with increased risk of cancer death. A clear example of this is the adipokine endotrophin (152), a cleavage product of the collagen VIα3 chain. Endotrophin has been shown to promote tumor growth by enhancing the ability of breast cancer cells to undergo epithelial to mesenchymal transition (EMT) in mice and humans (153). Interestingly, TZDs have been shown to decrease levels of endotrophin in obese patients (154).

A second exciting option to consider when considering the use of PPARγ ligands in cancer treatment is the role of the receptor in epithelial to mesenchymal transition (EMT). Epithelial cells that undergo EMT in the primary tumor acquire crucial features that increase their invasiveness, migratory phenotype, and resistance to apoptosis that are essential for the development of metastasis (155). Transdifferentiation of breast cancer epithelial cells undergoing EMT into post-mitotic adipocytes cells using TZDs and MEK inhibitors have been shown to be a promising therapeutic approach to repress primary tumor invasion and metastasis formation (156). The ability of PPARγ to drive or inhibit EMT might be subjected to the specific cell type from which the tumor arises however, as for example different studies in lung cancer cells have shown PPARγ ligands to inhibit and promote EMT (157). More research is needed to study the implication of PPARγ in EMT to fully determine its role and if it can be a real cancer treatment option.

PPARγ plays a pivotal role in the crossroad between obesity, immunity, and cancer. Understanding the common and unique molecular mechanism underlying the function of PPARγ in these situations will allow the development of new therapies. In order to do so, some challenges have to be overcome; achieving a selective modulation of PPARγ and a cell-specific delivery of these modulators are two of them. In order to maximize the beneficial effects of targeting PPARγ, the key might be that PPARγ has to be targeted in one specific cell type, and not indiscriminately throughout the whole body. The use of nanoparticles coupled to biological ligands that binds to specific membrane receptors for drug delivery is a technique that is been study for cancer treatment and it could have a bright future in the nuclear receptor field if its proven successful. Given the different and complex roles of PPARy in metabolism, immunity, and cancer, which rely on overlapping and diverse mechsmisms of action, cell-specific delivery of PPARγ ligands, especially noncanonical agonist ligands (NALs) and selective PPARy modulators (SPPARMs), represent a promising field of study for future research.

## Author Contributions

MH-Q, MB, and EK drafted, edited, and revised the manuscript. All authors contributed to the article and approved the submitted version.

## Funding

MH-Q was funded by the European Training Networks Project (H2020-MSCA-ITN-308 2016 721532).

## Conflict of Interest

The authors declare that the research was conducted in the absence of any commercial or financial relationships that could be construed as a potential conflict of interest.
